# A Minimally Invasive Open Approach With Ultrasound Guidance for Tibial Avulsion Fracture of the Posterior Cruciate Ligament: The Surgical Technique

**DOI:** 10.7759/cureus.84082

**Published:** 2025-05-14

**Authors:** Yusuke Kawanishi, Makoto Kobayashi, Hiroaki Fukushima, Hideki Murakami, Masahiro Nozaki

**Affiliations:** 1 Department of Orthopedic Surgery, Nagoya City University Graduate School of Medical Sciences, Nagoya, JPN

**Keywords:** avulsion fracture, orthopedic surgery, posterior cruciate ligament, surgical technique, ultrasound

## Abstract

Surgical intervention is generally recommended for a displaced tibial avulsion of the posterior cruciate ligament. However, there is still controversy regarding the optimal operative procedure, particularly between the conventional open approach and arthroscopic techniques.

We have developed a new, minimally invasive open approach with ultrasound guidance that allows for precise localization of neurovascular structures, reducing the risk of injury while maintaining clear access to the fracture site. We report the surgical technique and describe a case series of patients who were treated with this new method. This method is simpler and requires less technical expertise than the conventional open approach or arthroscopic techniques, thereby having the potential to be a safe and practical alternative for treating the tibial avulsion of the posterior cruciate ligament.

## Introduction

Tibial bony avulsions of the posterior cruciate ligament (PCL) are rare injuries that can lead to significant morbidity if not recognized and treated properly [1,2]. Although surgical intervention is generally recommended for displaced tibial avulsions, the optimal procedure has not yet been determined [2,3]. Traditionally, open reduction and internal fixation have been performed, with the medial-based surgical approaches described by Burks and Schaffer being the most popular [4-6]. The open approach allows direct visualization and reduction of bone fragments [2]. However, large incisions and release or excessive retraction of the posterior compartment muscles of the knee are needed [7]. Arthroscopic techniques have been reported and have the advantages of small incisions and the ability to confirm other intra-articular injuries [7-10]. However, this approach is technically demanding and has a considerable learning curve [7,10]. To resolve these problems, we have developed a new, minimally invasive and open approach with ultrasound guidance.

## Case presentation

Preoperative examination

The diagnosis of tibial avulsion of the PCL was confirmed by the patient’s history (most commonly, injuries when posterior-directed force is applied to the proximal tibia with the knee flexed) [2], physical examination, and diagnostic imaging. The operative criteria were the Meyers-McKeever II and III with > 3 mm of displacement of the bony fragment. This technique may not be applicable in cases with comminuted fractures or very small bone fragments that are challenging to fix with screws. Knee instabilities, such as posterior tibial sagging, were also assessed. Magnetic resonance imaging (MRI) was performed to evaluate the presence of intrasubstance PCL tears and other injuries, such as meniscus, ligament, and cartilage; if these injuries were suspected, additional procedures were considered.

Surgical technique

Ultrasound Guidance

**Figure 1:**
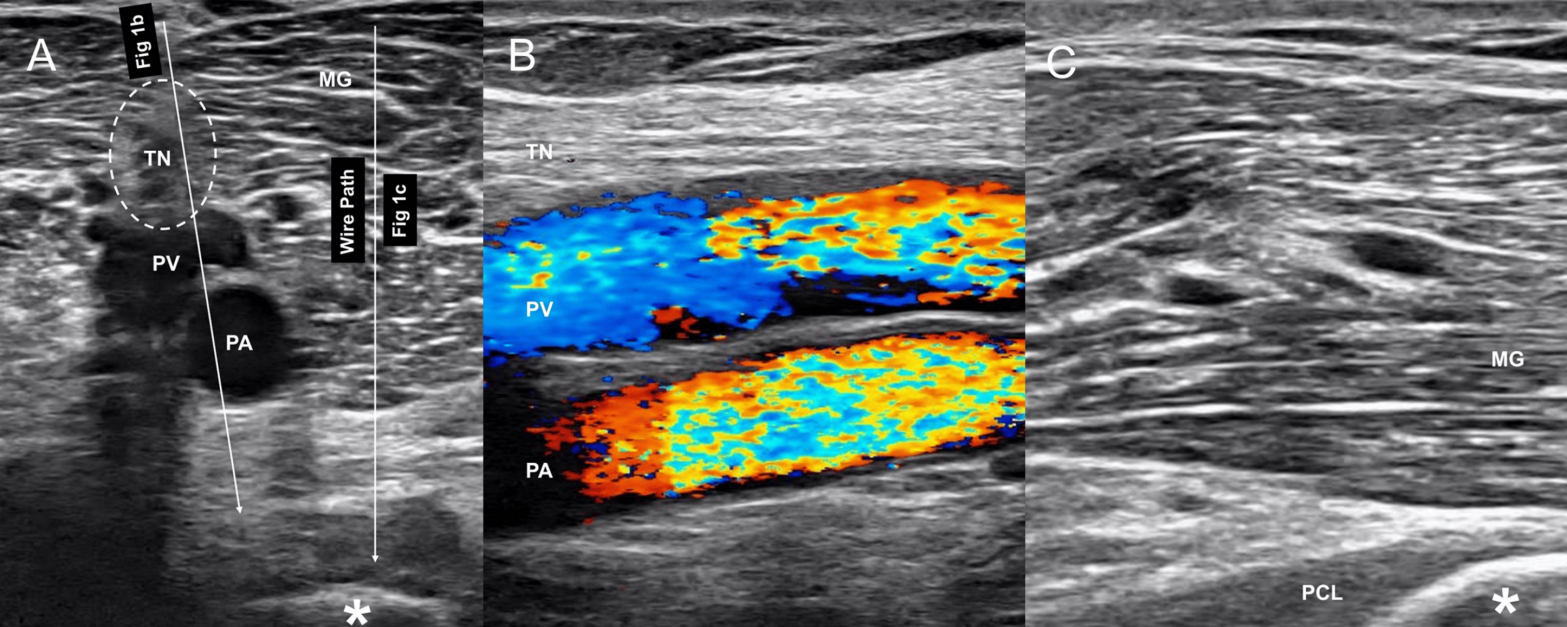
Pre-scan using ultrasound in the posteromedial knee (A) Kirschner wire is inserted through the MG toward the bony fragment (asterisk) as shown in the wire path. (B) The neurovascular bundle. (C) The appropriate location of the wire toward the bony fragment (asterisk). MG, medial head of the gastrocnemius muscle; PCL, posterior cruciate ligament; PA, popliteal artery; PV, popliteal vein; TN, tibial nerve.

**Figure 2:**
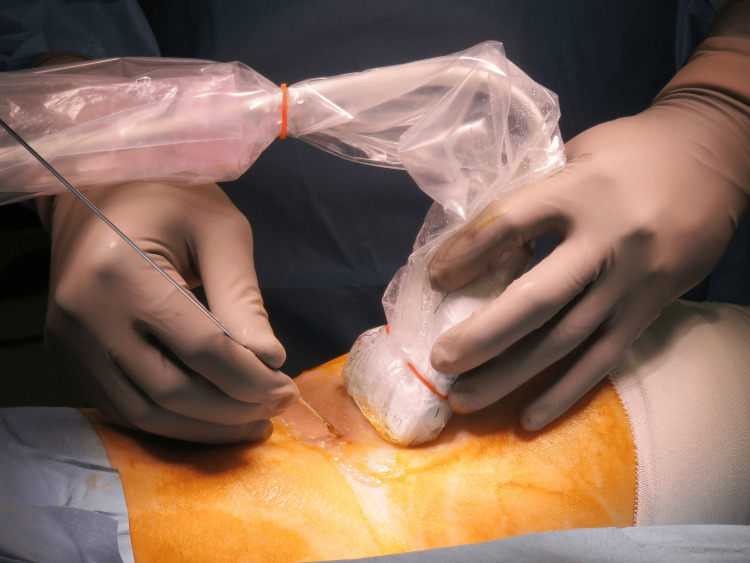
Insertion of a Kirschner wire

**Figure 3:**
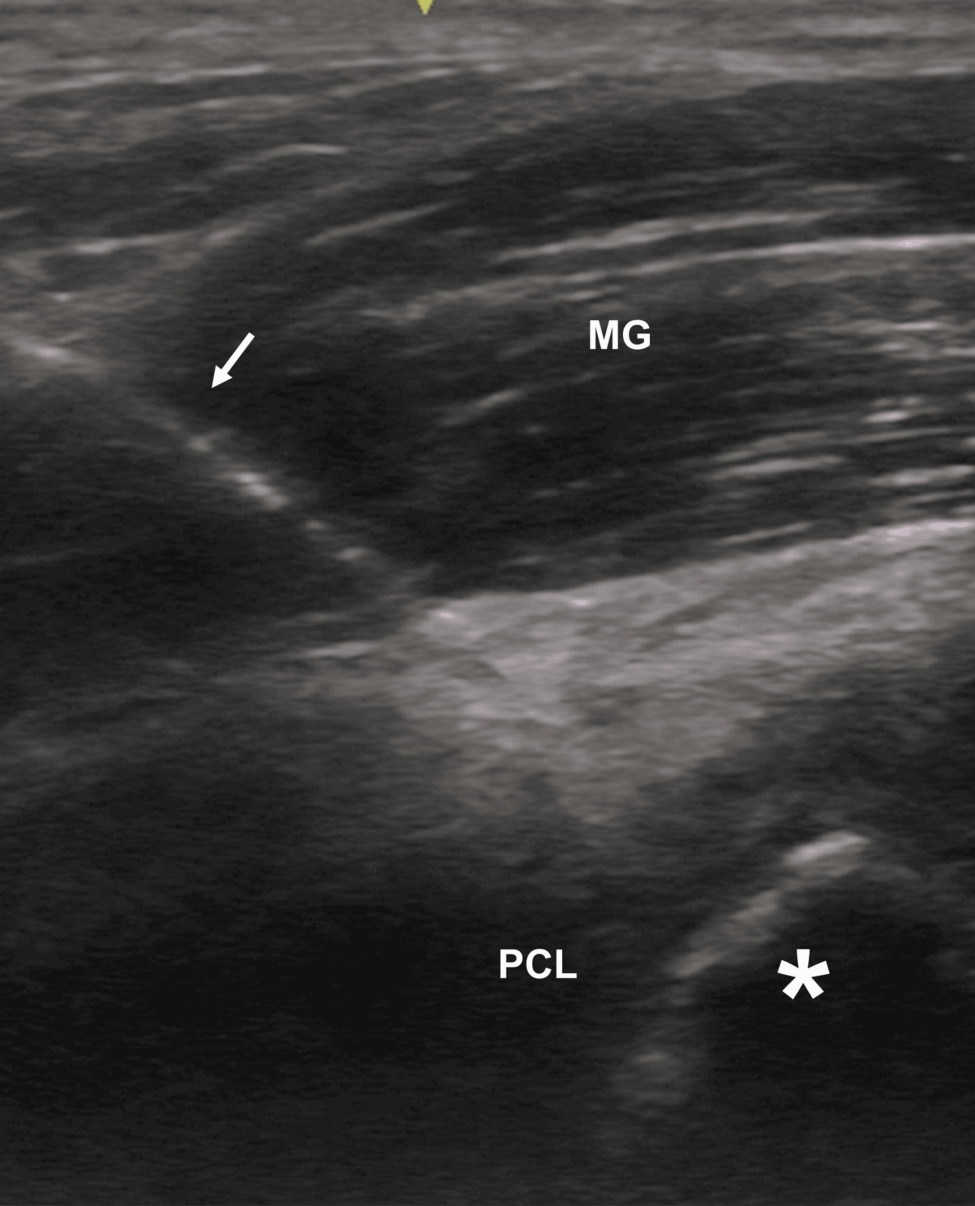
An ultrasound image while Kirschner wire is being inserted Kirschner wire (arrow) is inserted to the bony fragment (asterisk). MG, medial head of the gastrocnemius muscle; PCL, posterior cruciate ligament

Surgery was performed under spinal or general anesthesia in the prone position. A tourniquet was applied over the ipsilateral thigh depending on the surgeon’s preference. First, a pre-scan was performed using ultrasonography to identify the neurovascular bundle, fracture fragment, medial head of the gastrocnemius (MG), and PCL in both short- and long-axis views (Figure 1). A Kirschner wire was percutaneously inserted under ultrasound guidance as a reference for the open approach (Figure 2). The wire was inserted through the MG toward the bone fragment, ensuring that there was sufficient distance from the neurovascular bundle in both the short- and long-axis views (Figures 1-3).

Fluoroscopic Confirmation

**Figure 4:**
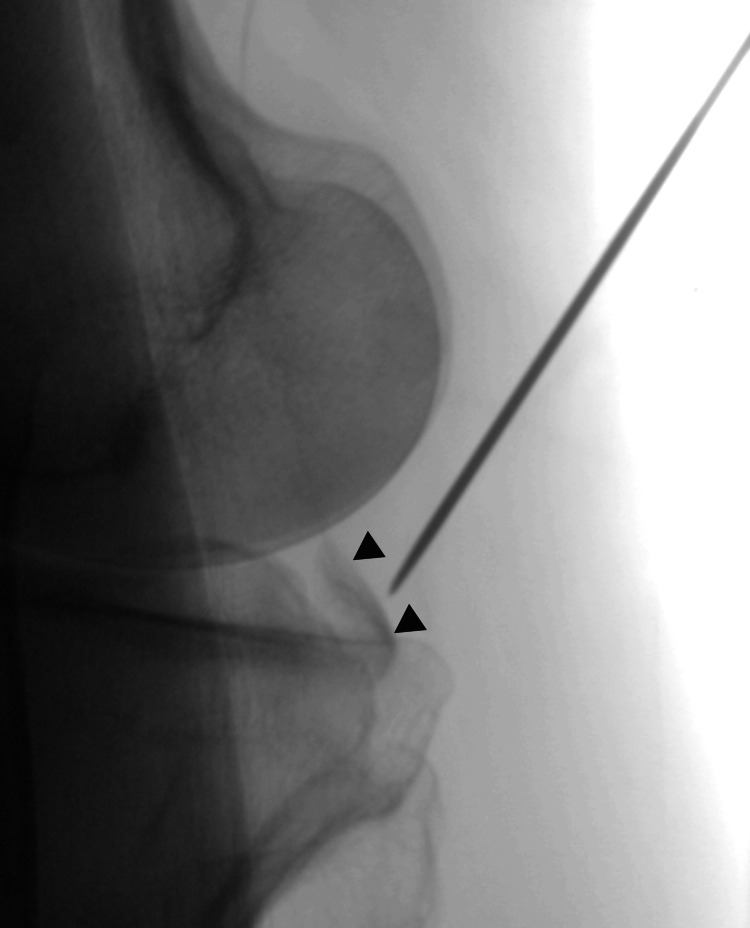
Fluoroscopic evaluation of the wire path Fluoroscopic evaluation was performed to confirm the trajectory of the wire toward the bony fragment (arrowheads).

**Figure 5:**
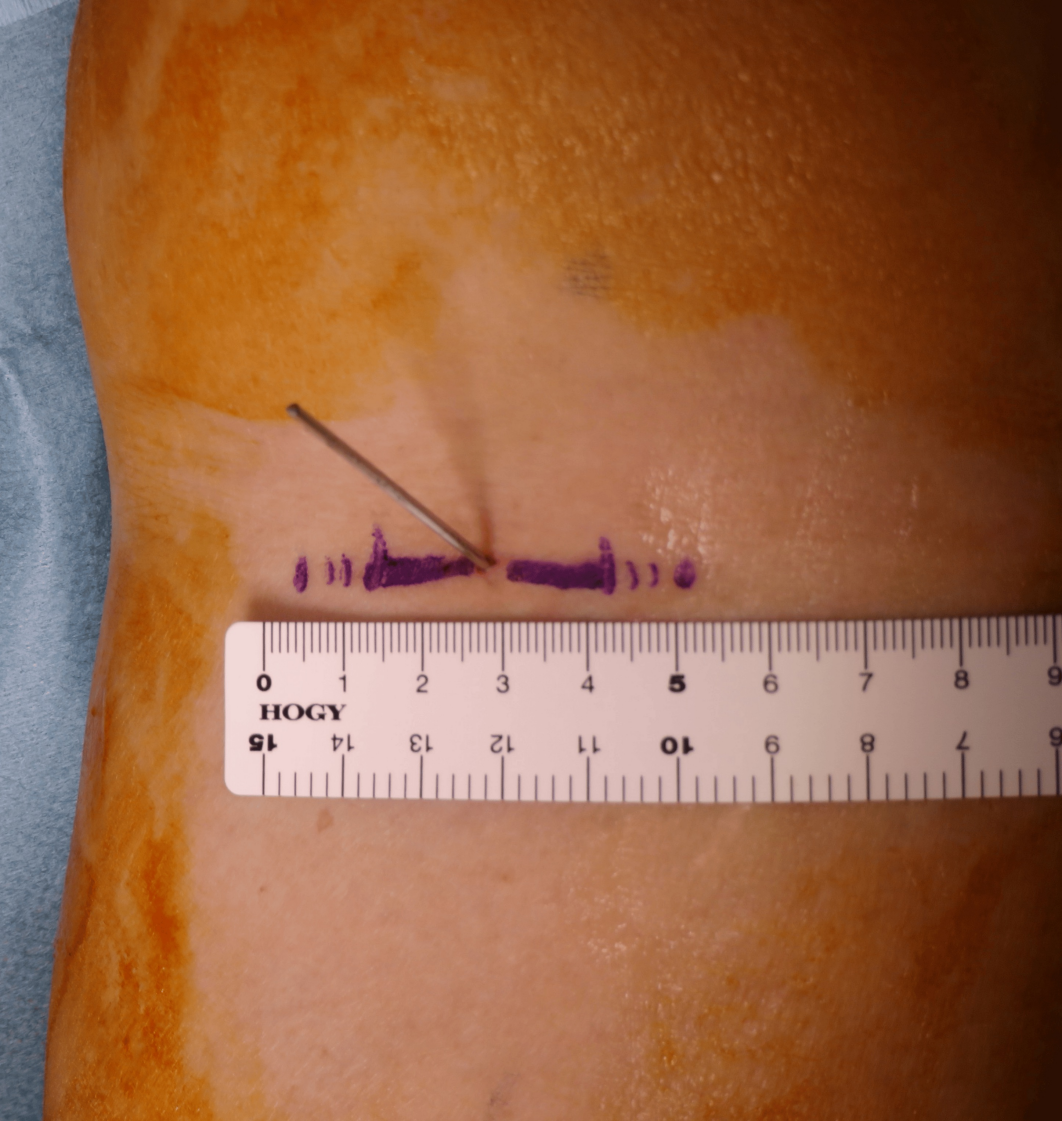
Horizontal incision

Fluoroscopic confirmation was necessary because it was difficult to macroscopically confirm the position and angle of the wire using ultrasound alone (Figure 4). The angle and position of wire insertion should be corrected accordingly. When the angle and position were adjusted appropriately, a 3-5 cm horizontal skin incision was made at the wire insertion point (Figure 5). The incision was often located on the proximal popliteal crease.

Dissection and Fracture Fixation

**Figure 6:**
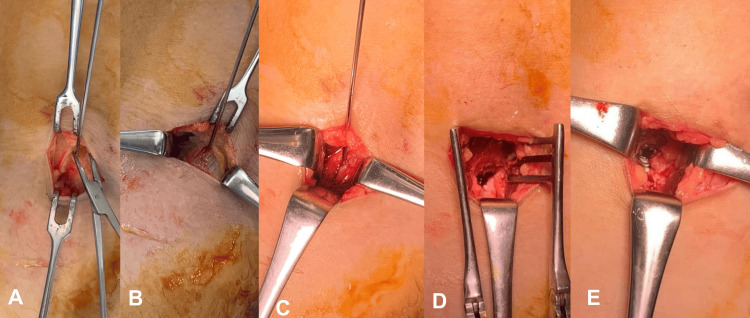
Blunt dissection to the bony fragment (A) The blunt dissection is performed along the wire. (B, C) The medial head of the gastrocnemius (MG) is split. (D) The MG is retracted. (E) A screw is inserted.

After the fascia of the MG was incised, blunt dissection was performed along the wire to the bone fragment and fracture bed. This procedure can be performed safely because ultrasound views confirmed that this wire is medially free from the neurovascular bundle and reaches the bone fragments (Figure 6A). The MG was split and the lateral half was retracted laterally, protecting the neurovascular bundle in the popliteal space (Figure 6B-6D) [4]. The posterior capsule was incised and the fracture site was identified. After the reduction of the fracture fragment, fixation was performed with cannulated cancellous screws (Figure 6E). The pitfalls of this surgical procedure are listed in Table 1. The advantages and disadvantages of this technique are listed in Table 2.

**Table 1:** Pitfalls of minimally invasive open approach with ultrasound guidance

Pitfalls	
1.	After wire insertion with ultrasound-guidance, fluoroscopy should be used to confirm the trajectory of the wire because the wire is often too parallel to the articular surface of the tibia and is positioned too distal.
2.	The horizontal skin incision makes it difficult to adjust the distal-proximal position. Ensure that the point of skin incision is not too distal and is at the proximal popliteal crease.
3.	A deep retractor, such as a self-retaining retractor for spinal surgery, is useful for dissection.
4.	Confirmation with ultrasound should be done before subcutaneous dissection because after dissection, confirmation is difficult due to the presence of air.

**Table 2:** Advantages and disadvantages of minimally invasive open approach with ultrasound guidance

Advantages		Disadvantages	
1.	Less invasive than conventional open approach.	1.	Limited surgical field of view.
2.	The procedure can be performed with the knee extended because there is no need to loosen and retract the entire medial head of the gastrocnemius muscle. This leads to loosening of the PCL and easy reduction of the bone fragment.	2.	It is difficult in cases where the bone fragment is comminuted or too small to be fixed with screws.
3.	Easy to reach the fracture site.	3.	If the neurovascular bundle is positioned medially, it is difficult to insert the Kirschner wire.
4.	Less technically demanding than arthroscopic surgery.		
5.	Low risk of neurovascular injury because the location of the Kirschner wire, free of the neurovascular bundle, has already been confirmed using ultrasound and because the lateral half of the medial head of the gastrocnemius muscle protects the neurovascular bundle.		

Postoperative Management

During the first postoperative week, patients were restricted to knee flexion from 0° to 30° and allowed to bear half their weight with extension bracing. For one to eight weeks, the patients performed full weight-bearing with a posterior-support knee brace and were limited to 120° of knee flexion. After bony union was achieved, the restrictions were removed according to tolerance.

Case series

Case 1

**Figure 7:**
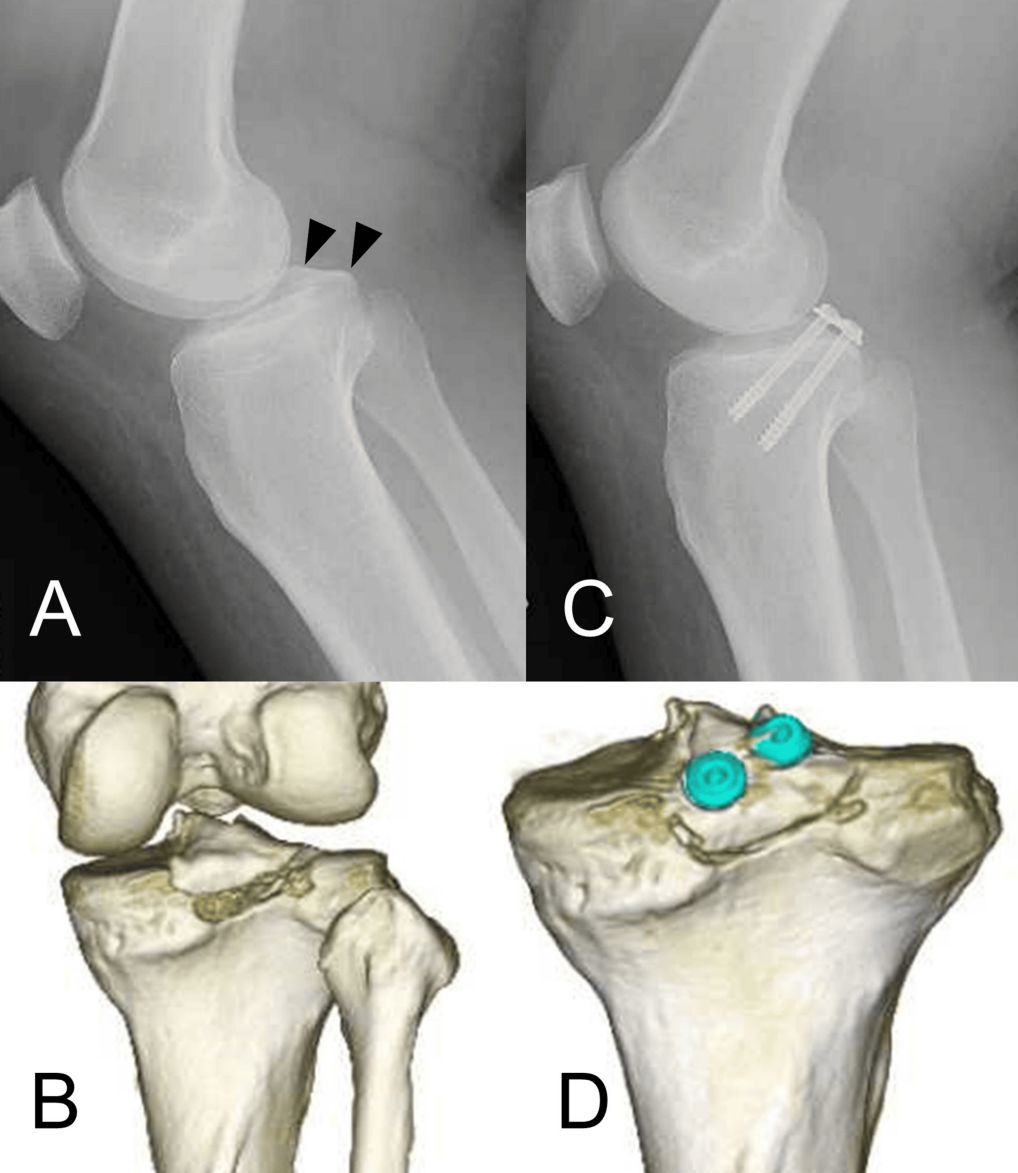
Case 1 (A) Preoperative lateral radiograph (arrowhead: bony fragment), (B) CT image. (C) Postoperative lateral radiograph and (D) CT image. CT, computed tomography; MRI, magnetic resonance imaging.

A 48-year-old woman (weight, 99 kg; length, 157 cm) was injured by bruising her right knee due to a fall. Preoperative lateral radiograph and CT scan showed the bony fragment that was displaced 6 mm (Figure 7A, 7B). Surgery was performed 12 days after the injury (Figure 7C, 7D). Operative time and blood loss were 103 minutes and 5 ml, respectively. The patient was discharged 12 days after surgery. At three months, bone union was achieved. At four months, the patient returned to her previous job with knee extension of 0° and flexion of 130°. At nine months, the patient had neither complications nor posterior tibial sagging; Lysholm score: 88, Knee Injury and Osteoarthritis Outcome Score (KOOS): 84.

Case 2

**Figure 8:**
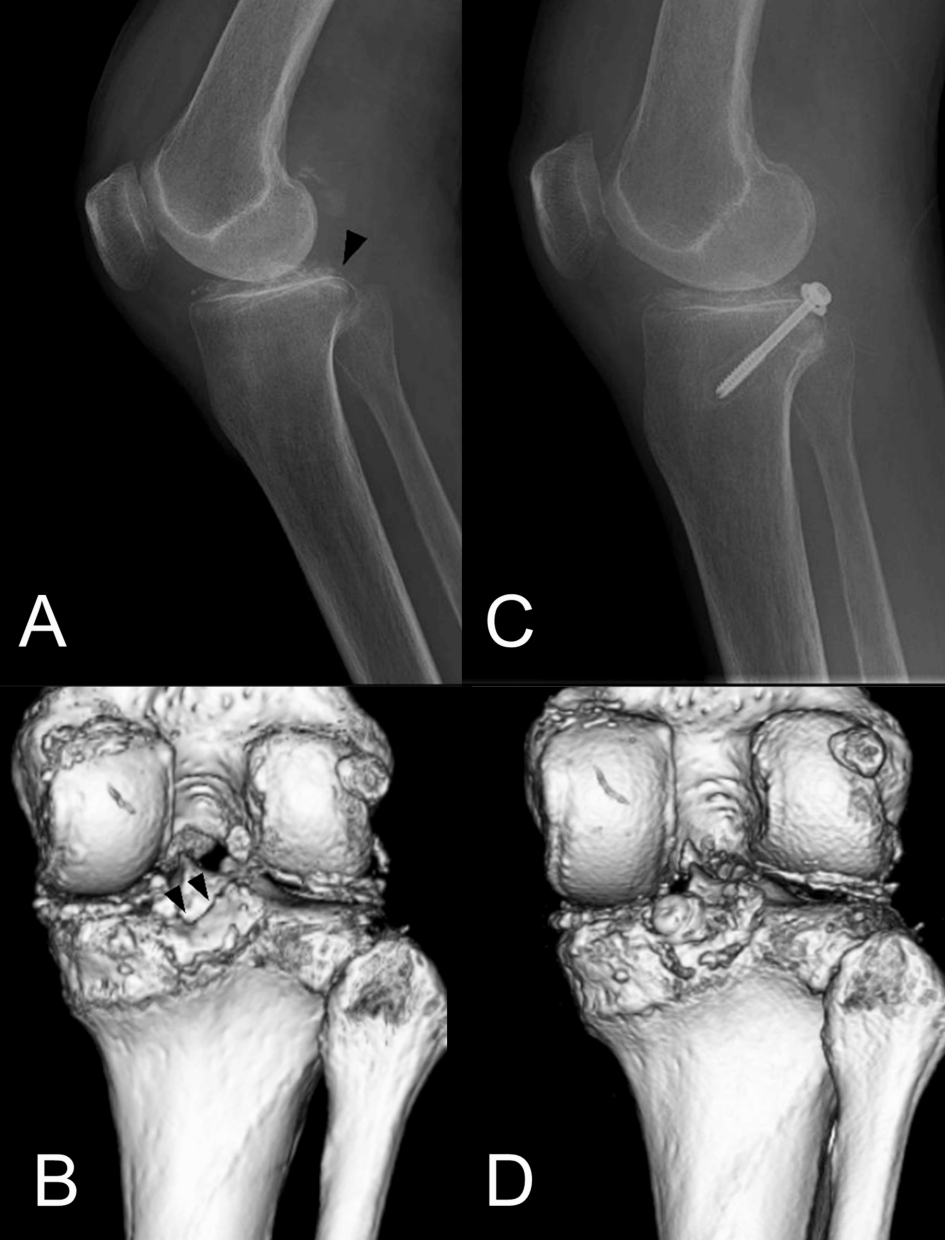
Case 2 (A) Preoperative lateral radiograph (arrowhead: bony fragment), (B) CT image (arrowhead: bony fragment). (C) Postoperative lateral radiograph and (D) CT image. CT, computed tomography; MRI, magnetic resonance imaging.

An 83-year-old woman (weight, 40 kg; length, 155 cm) was injured by bruising her right knee due to a fall. Preoperative lateral radiograph and CT scan showed the bony fragment that was displaced 4 mm (Figure 8A, 8B). Surgery was performed two days after the injury (Figure 8C, 8D). Operative time and blood loss were 67 minutes and minimal, respectively. The patient was discharged seven days after surgery. At one month, bone union was achieved. At two months, the patient returned to her daily activities with no restrictions at all and could sit on her heels with her knees flexed; knee extension of 0° and flexion of 150°. At 13 months, the patient had no knee symptoms at all and neither complications nor posterior tibial sagging; Lysholm score: 100.

Case 3

**Figure 9:**
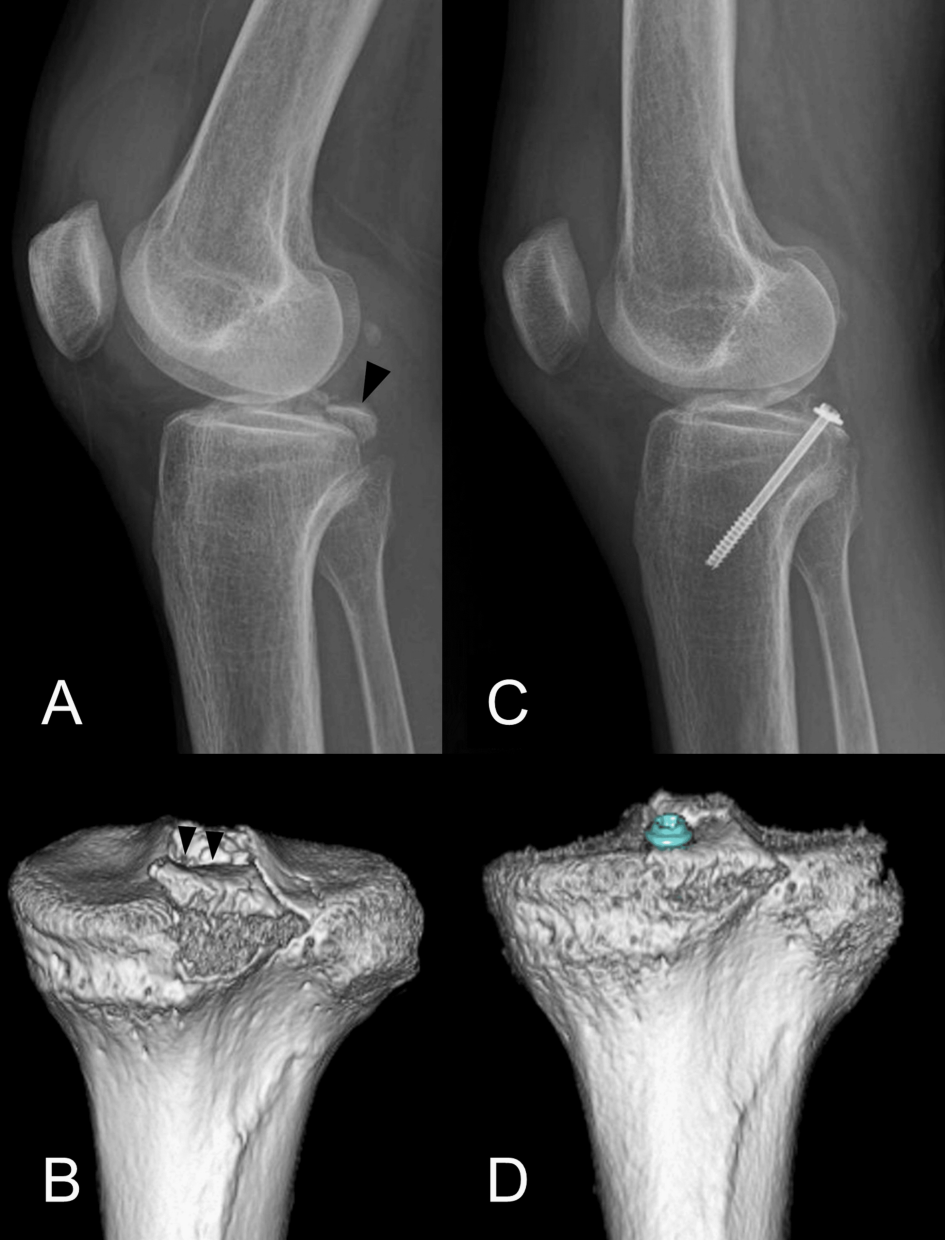
Case 3 (A) Preoperative lateral radiograph (arrowhead: bony fragment), (B) CT image (arrowhead: bony fragment). (C) Postoperative lateral radiograph and (D) CT image. CT, computed tomography; MRI, magnetic resonance imaging.

A 55-year-old woman (weight, 44 kg; length, 156 cm) was injured with a bruised knee due to a fall. Preoperative lateral radiograph and CT scan showed the bony fragment that was displaced 7 mm (Figure 9A, 9B). Surgery was performed six days after the injury (Figure 9C, 9D). Operative time and blood loss were 29 minutes and minimal, respectively. The patient was discharged five days after surgery. At three months, bone union was achieved. At four months, the patient returned to her daily activities with no restrictions at all; knee extension of 0° and flexion of 140°. At 13 months, the patient had neither complications nor posterior tibial sagging; Lysholm score: 87, KOOS: 90.

## Discussion

A systematic review of PCL avulsions [8] reported that there was no noticeable difference between the open and arthroscopic approach groups in patient-reported outcome measures at the final follow-up, and both studies on the open and arthroscopic approaches showed satisfactory outcomes. Despite arthroscopic techniques having a long learning curve [7,10], surgery for this fracture is infrequently performed in most institutions [1,2]. Therefore, a minimally invasive and simple approach is beneficial. In the present technique, a pre-scan was performed to confirm the appropriate position of skin incision, and a deep retractor was used for exposing the fracture site, which greatly shortened the operative time, as seen in Case 3. Therefore, it is possible that the learning curve is short for this technique, but more cases need to be evaluated to confirm this.

To date, only a few minimally invasive open approaches have been reported. Zhao et al. [6] reported an approach using a horizontal incision in which the gastrocnemius and semitendinosus were dissected with knee flexion at 30°, and the MG was pulled laterally. They reported that the intraoperative bleeding and pain scores were significantly better than those of the traditional L-shaped incision group. However, it required knee flexion and did not allow neurovascular localization. Gavaskar et al. [7] reported an approach utilizing the interval between the two heads of the gastrocnemius in which a transverse incision was made using fluoroscopy. However, this technique required a direct neurovascular retraction. The present ultrasound-guided method can be performed with knee extension and may have a lower risk of neurovascular injuries because the neurovascular bundle is identified in advance using ultrasound, and no direct retraction to the neurovascular bundle is performed. In these case series, the intraoperative blood loss was very low, no complications were observed, and the patient returned to work relatively early, with good functional results.

This case series and the surgical techniques have some limitations. First, the findings are based on a few cases, which restricts their applicability. A more extensive series of cases or a comparative study at long-term follow-up is needed for robust conclusions about this technique. Second, the reproducibility of ultrasound technique may be limited in settings where surgeons are less familiar with ultrasonography. Third, this technique requires the use of fluoroscopy at the pre-scan and intra-operation. Therefore, radiation exposure is unavoidable. However, no matter which approach is used, the use of fluoroscopy is often required for screw fixation. Furthermore, compared to the traditional open approach, this approach only requires an additional moment of pre-scan and does not cause a large amount of radiation exposure.

## Conclusions

The described technique presents a minimally invasive open approach for treating tibial avulsion fractures of the PCL, using ultrasound guidance. Ultrasound allows for precise localization of neurovascular structures, reducing the risk of injury while maintaining clear access to the fracture site.

This method is simpler and requires less technical expertise than traditional open or arthroscopic techniques, making it more accessible for general orthopedic practice. Although further studies are needed, it shows promise as a safe and practical alternative for treating PCL avulsion fractures.
